# Lung Transplantation in a Patient with COPA Syndrome

**DOI:** 10.1155/2020/3624795

**Published:** 2020-03-31

**Authors:** Jorge M. Mallea, Anna Kornafeld, Andras Khoor, David B. Erasmus

**Affiliations:** Mayo Clinic, 4500 San Pablo Rd S, Jacksonville, FL 32224, USA

## Abstract

COPA syndrome is a newly discovered, rare genetic autoimmune disorder, which can affect the lungs, joints, and kidneys. It is difficult to recognize, and the survival benefit of lung transplantation for these patients is not yet known. We present a case of a 24-year-old woman who received bilateral lung transplant for COPA syndrome. At 15 months posttransplant, her pulmonary function is stable with no episodes of acute cellular- or antibody-mediated rejection and no evidence of disease recurrence.

## 1. Introduction

COPA syndrome was first reported in 2015 [1]. It is a rare, genetic autoinflammatory disorder, which can affect the lungs, joints, and kidneys. It is caused by mutations in the gene of coatomer subunit *α*, a subunit of coat protein complex I (COPI). COPI is a carrier complex involved in the intracellular retrograde protein transport from Golgi to endoplasmic reticulum. The product of the mutant gene triggers processes that play a role in developing autoimmunity [1]. Since it is a rare disease, COPA syndrome is difficult to recognize. The following common pulmonary findings have been described in genetically confirmed cases: cysts, follicular bronchiolitis, and diffuse alveolar hemorrhage. Other common characteristics include positive family history, early age onset, COPA syndrome in another family member, arthritis, nephritis, and positivity for antineutrophil cytoplasmic antibody (ANCA), antinuclear antibody (ANA), or rheumatoid factor (RF) [2].

## 2. Case Presentation

**Figure 1:**
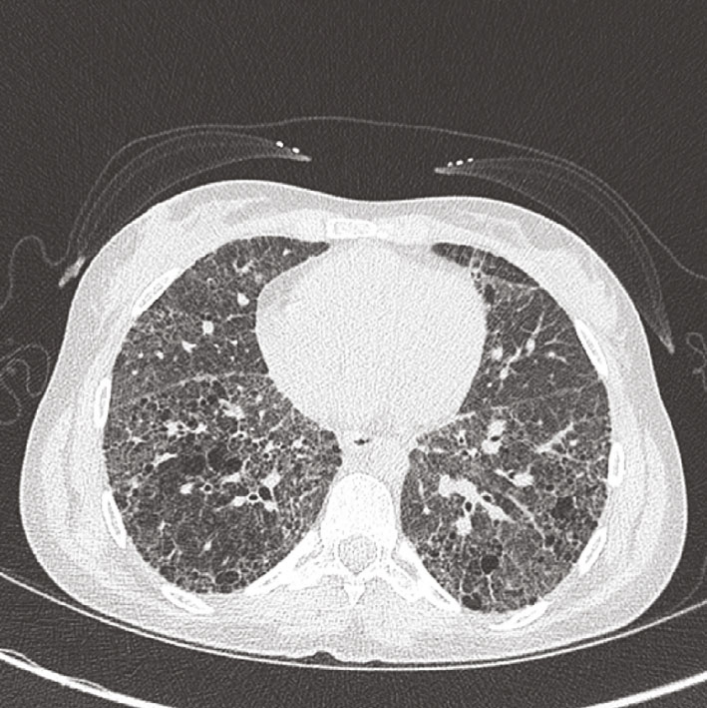
Chest computed tomography of the patient's native lungs demonstrating cystic changes, diffuse coarse reticular interstitial thickening with associated ground glass opacities.

**Figure 2:**
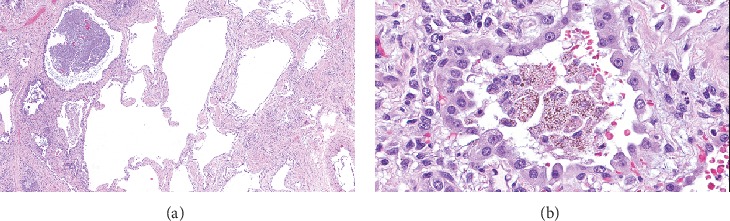
Histologic sections of the explanted (native) lungs show (a) fibrosing interstitial pneumonia with airspace enlargement (hematoxylin-eosin, original magnification 30x) and (b) alveolar hemorrhage with hemosiderin-laden macrophages and reactive type 2 cells (hematoxylin-eosin, original magnification 400x).

A 24-year-old woman received bilateral lung transplant for COPA syndrome. Manifestations of her COPA syndrome included arthritis, biopsy-confirmed immune-mediated kidney disease, antineutrophil cytoplasmic antibody (ANCA) positivity, interstitial lung disease, diffuse alveolar hemorrhage, and progressive respiratory failure leading to mechanical ventilator support. Chest computed tomography demonstrated extensive cystic changes in the lungs, diffuse coarse reticular interstitial thickening, and ground glass opacities (Figure 1). Pulmonary function tests showed a restrictive pattern with decreased DLCO. She received rituximab and plasmapheresis in the perioperative period and induction immunosuppression with thymoglobulin. Histologic sections of the explanted lungs showed a relatively uniform fibrosing interstitial pneumonia (nonusual interstitial pneumonia pattern) with honeycomb change and emphysema (Figure 2). Organizing lung injury and patchy alveolar hemorrhage with hemosiderin-laden macrophages were noted, but capillaritis was not seen.

The postoperative course was complicated by neutropenic fever, cytomegalovirus viremia, bilateral lymphocytic pleural effusions, and persisting leukopenia. A year after transplant, she had no respiratory symptoms, but testing confirmed severe gastroesophageal reflux disease (GERD). She was successfully treated with laparoscopic Nissen fundoplication. At 15 months posttransplant, her pulmonary function is stable, with no episodes of acute cellular- or antibody-mediated rejection and no evidence of disease recurrence. She is maintained on an immunosuppression regimen that includes prednisone, tacrolimus, and intravenous immunoglobulin.

## 3. Discussion

The survival benefit of lung transplantation for patients with COPA syndrome is not yet known. In general, immune dysregulation associated with autoimmune disorders may be a reason for concern. According to a recent study, patients who underwent lung transplantation for nonscleroderma connective tissue-related lung disease are not more likely to develop acute rejection or bronchiolitis obliterans syndrome compared with IPF [3]. To our knowledge, only three other adult COPA syndrome patients have received lung transplants [4, 5]. One had a “near standard” posttransplant survival; survival of the other two patients (mother and adult son) has not been reported. Our patient is doing well 15 months after transplantation, but an international registry is needed to clarify the outcome. In the meantime, transplantation of COPA syndrome patients requires careful consideration.

## Abbreviations

ANA: Antinuclear antibody
ANCA: Antineutrophil cytoplasmic antibody
COPA: Coatomer subunit alpha
COPI: Coat protein complex I
DLCO: Diffusing capacity of the lungs for carbon monoxide
GERD: Gastroesophageal reflux disease
RF: Rheumatoid factor.
